# Iodine-125 seed represses the growth and facilitates the apoptosis of colorectal cancer cells by suppressing the methylation of miR-615 promoter

**DOI:** 10.1186/s12885-021-09141-4

**Published:** 2022-01-08

**Authors:** Fenghai Ren, Baojun Li, Chao Wang, Yanbo Wang, Binbin Cui

**Affiliations:** 1 Department of Thoracic Surgery, Harbin Medical University Cancer Hospital, Harbin, 150081 Heilongjiang China; 2 Department of Head and Neck Surgery, Harbin Medical University Cancer Hospital, Harbin, 150081 Heilongjiang China; 3 Department of Prenatal Diagnosis, The 2nd Affiliated Hospital of Harbin Medical University, Harbin, 150081 Heilongjiang China; 4 Department of Colorectal Surgery, Harbin Medical University Cancer Hospital, 150 Haping Road, Harbin, 150081 Heilongjiang China

**Keywords:** Iodine-125 seed, microR-615, Methylation, Colorectal cancer, 5-AZA, MAPK pathway

## Abstract

**Background:**

Colorectal cancer (CRC) represents a common malignancy in gastrointestinal tract. Iodine-125 (^125^I) seed implantation is an emerging treatment technology for unresectable tumors. This study investigated the mechanism of ^125^I seed in the function of CRC cells.

**Methods:**

The CRC cells were irradiated with different doses of ^125^I seed (0.4, 0.6 and 0.8 mCi). miR-615 expression in CRC tissues and adjacent tissues was detected by RT-qPCR. miR-615 expression was intervened with miR-615 mimic or miR-615 inhibitor, and then the CRC cells were treated with 5-AZA (methylation inhibitor). The CRC cell growth, invasion and apoptosis were measured. The methylation level of miR-615 promoter region was detected. The xenograft tumor model irradiated by ^125^I seed was established in nude mice. The methylation of miR-615, Ki67 expression and CRC cell apoptosis were detected.

**Results:**

^125^I seed irradiation repressed the growth and facilitated apoptosis of CRC cells in a dose-dependent manner. Compared with adjacent tissues, miR-615 expression in CRC tissues was downregulated and miR-615 was poorly expressed in CRC cells. Overexpression of miR-615 suppressed the growth of CRC cells. ^125^I seed-irradiated CRC cells showed increased miR-615 expression, reduced growth rate and enhanced apoptosis. The methylation level of miR-615 promoter region in CRC cells was decreased after ^125^I seed treatment. *In vivo* experiments confirmed that ^125^I seed-irradiated xenograft tumors showed reduced methylation of the miR-615 promoter and increased miR-615 expression, as well as decreased Ki67 expression and enhanced apoptosis. The target genes of miR-615 and its regulatory downstream pathway were further predicted by bioinformatics analysis.

**Conclusions:**

^125^I seed repressed the growth and facilitated the apoptosis of CRC cells by suppressing the methylation of the miR-615 promoter and thus activating miR-615 expression. The possible mechanism was that miR-615-5p targeted MAPK13, thus affecting the MAPK pathway and the progression of CRC.

**Supplementary Information:**

The online version contains supplementary material available at 10.1186/s12885-021-09141-4.

## Introduction

Colorectal cancer (CRC) remains the dominant cause of cancer-associated deaths, despite considerable advances in diagnosis and treatment [1]. The onset of CRC is attributed to genetic and epigenetic changes, which results in homeostasis dysfunction and neoplastic transformation [2]. Age, genetic and environmental factors are widely involved in the initiation of CRC; other recognized risk factors include inflammatory bowel disease, obesity, sedentary lifestyle, history of abdominal radiation and acromegaly [3]. Preoperative chemoradiotherapy concomitantly with 5-fluorouracil is accepted as the standard treatment for CRC [4]. However, approximately half of CRC patients may develop distant metastasis or recurrence, which eventually leads to systemic control failure and unfavorable outcome [5]. Recently, endorectal brachytherapy has emerged as a viable neoadjuvant treatment option for locally advanced or recurrent CRC [6, 7]. Hence, it is of great clinical significance to elucidate the potential molecular mechanism of brachytherapy for CRC.

Iodine-125 (^125^I) seed implantation constitutes a type of brachytherapy [8], which has been extensively applied in clinical tumor treatment due to its advantages of high precision, minimal trauma, potent lethality and few complication [9]. Percutaneous computed tomography-guided ^125^I seed implantation represents a minimally invasive treatment technique for the CRC patients with lung metastases [10]. Radioimmunoassay-guided surgery for CRC patients using ^125^I seed-labeled anticarcinoembryonic antigen antibody can enable the surgeons to identify lymph node metastasis, thereby ensuring the personalized radical operation [11]. Despite the wide clinical practice of ^125^I seed implantation, its radiobiological effects and the potential molecular mechanism are not thoroughly clarified yet.

Emerging evidence has revealed that the irradiation-triggered DNA demethylation may play a vital role in the therapeutic effect of ^125^I seed [12]. DNA methylation participates in numerous crucial cellular processes, such as cell cycle, signal transduction and angiogenesis [13]. The irradiation-triggered DNA demethylation contributes to repressing tumor progression by activating tumor suppressor microRNA (miRNA) genes [12]. miRNAs are small noncoding RNA molecules consisting of 18–23 nucleotides, which affects gene silencing and translational repression via binding to target mRNAs [14]. miRNA-based therapy, whether restoring or suppressing its expression and activity, holds great promise for the treatment of human malignancies [15]. miR-615, a highly conserved miRNA in mammals, is not only implicated in embryogenesis, but also in the modulation of cell growth, proliferation and migration [16]. miR-615 is demonstrated to function as a tumor suppressor in glioblastoma [17], prostate cancer [18] and osteosarcoma [19]. miR-615-5p is aberrantly downregulated in pancreatic ductal adenocarcinoma cells because of promoter hypermethylation, thus inducing the tumor cell growth and invasion [20]. Elevated miR-615-3p expression is concerned with the tumors in the right colon [21]. Nevertheless, the exact role of miR-615 in CRC needs further investigation. This study sought to determine whether miR-615 expression can be modulated by irradiation-induced DNA demethylation and implicated in ^125^I seed-triggered tumor inhibition in CRC, which shall confer a novel theoretical basis for the application of ^125^I seed implantation in CRC.

## Materials and methods

### Ethics statement

This study was approved by the Ethics Committee of Harbin Medical University Cancer Hospital, following the *Declaration of Helsinki*. The informed consent was obtained from each eligible participant. The animals were treated in accordance with the standards of animal ethics.

### Clinical samples

Twenty-seven CRC patients diagnosed in Harbin Medical University Cancer Hospital from August 2018 to August 2019 were recruited and the clinical characteristics of patients were shown in Supplementary Table 1. None of the patients received radiotherapy or preoperative chemotherapy. The tumor tissues and adjacent tissues were preserved in liquid nitrogen for subsequent analysis.

### Cell culture and grouping

SW480 cells (CCL-228, American Type Culture Collection, Manassas, VA, USA), HCT-8 cells (CCL-244) and immortalized normal intestinal epithelial cells (NCM460 cells) were cultured in Dulbecco’s modified Eagle’s medium (DMEM; Solarbio Science & Technology Co., Ltd., Beijing, China) containing 10% fetal bovine serum (FBS) at 37 °C with 5% CO_2_. After the cells adhered to the wall, they were passaged and detached with 0.25% trypsin (Hyclone Company, Logan, UT, USA). The cells at logarithmic phase were used for the experiments.

^125^I seed irradiation: internal ^125^I seeds were obtained from HTA Co., Ltd. (Beijing, China). The ^125^I seeds at the doses of 0.4 mCi (14.53 MBq), 0.6 mCi (22.97 MBq) and 0.8 mCi (29.97 MBq) were used for experiments. The *in vitro* irradiation device was established by using ^125^I seed irradiation model. Human colon cancer cells (SW480 and HCT-8) were irradiated by different doses of ^125^I seeds for 72 h. The total radiation doses were 113 cGy, 162 cGy and 225 cGy, respectively. The control cell lines were not irradiated by ^125^I seeds in the same device. The *in vitro* irradiation model was established as described previously [22]. The specific operation was as follows: in the *in vitro* model, eight ^125^I seeds were evenly wound on the circumference of 30 mm in diameter, and one ^125^I seed was placed in the center to process the cells.

Cell grouping: mimic NC, miR-615 mimic, inhibitor NC and miR-615 inhibitor were purchased from GenePharma (Shanghai, China). The cell transfection was performed using the Lipofectamine 2000 (Invitrogen Inc., Carlsbad, CA, USA), with the final concentration of 100 nm. After transfection, the cells were collected to evaluate the transfection efficiency. Thereafter, the transfected cells were irradiated by corresponding dose of ^125^I seed for subsequent experiments.

Solvent treatment grouping: dimethyl sulphoxide (DMSO) group and 5-Aza group (DNA methyltransferase inhibitor; Sigma-Aldrich, Merck KGaA, Darmstadt, Germany). After 24 h of culture, the cells adhered to the wall and then were placed in the 5-Aza (final concentration was 2 μmol/L) or DMSO (final concentration was 2 μmol/L) culture fluids. The culture fluid was refreshed every 24 h [23].

### 3-(4,5-dimethylthiazol-2-yl)-2,5-diphenyltetrazolium bromide (MTT) assay

The cell viability was determined by measuring the ability of cells to convert thiazolyl blue tetrazolium bromide (CT02, Sigma-Aldrich) into blue violet crystalline methyl tetrazolium (formazan). Briefly, 20 μL MTT solution [5 mg/mL in phosphate-buffered saline (PBS)] was added into the 96-well plates and cultured with cells for 5 h. Then, the medium was replaced by DMSO (200 μL/well). The optical density (OD) value at 570 nm was measured by a microplate reader (VSERSA Max, Molecular Devices, CA, USA).

### Colony formation assay

Briefly, 1.2% agar was heated and dissolved, and placed in 46 °C water bath for standby. The prepared CRC cells were counted and suspended in DMEM preheated at 40 °C. The 6-well plates were added with cell suspension (containing 1 × 10^3^ cells) per well. Then, 125 μL preheated 1.2% agar was gently and rapidly mixed with the above cell suspension and sample in the 6-well plates, avoiding bubbles. After natural solidification, the mixture was put in the incubator at 37 °C with 5% CO_2_. After 8–10 days, the cell colonies were stained with 0.1% crystal violet (Sigma-Aldrich, Dorset, USA) and observed and counted under the inverted microscope.

### Transwell assay

CRC cells were starved in serum-free medium for 24 h, then detached and washed with PBS twice. The cells were resuspended in serum-free Opti-MEMI (Invitrogen) containing 10 g/L bovine serum albumin (BSA; Sigma-Aldrich), and the cell density was adjusted to 3 × 10^4^ cells/mL. This experiment used 8 μm 24-well Transwell chamber (Corning Glass Works, Corning, NY, USA). Before the experiment, each chamber was coated with 50 μL Matrigel (Sigma-Aldrich), with 3 chambers in each group and 100 μL cell suspension in each chamber. The basolateral chamber was added with 600 μL 10% RPMI1640 medium and incubated at 37 °C with 5% CO_2_. After 48 h, the cells were fixed with 4% paraformaldehyde for 30 min, treated with 0.2% Triton X-100 (Sigma-Aldrich) for 15 min and stained with 0.05% gentian violet for 5 min. The number of stained cells was counted under the inverted microscope (Leica DMi8-M, Germany). Five visual fields were randomly selected. The experiment was repeated for three times.

### Flow cytometry

For apoptosis detection, 1 × 10^6^ cells at logarithmic phase were collected and washed with cold PBS twice. The cells were suspended in 1 × Annexin buffer, added with 5 μL Annexin-VFITC (Becton Dickinson Bio-sciences) and placed in the dark at room temperature for 10 min. After mixing, the cells were placed in the dark at room temperature for 5 min and washed with cold PBS once. The cells were suspended in 300 μL 1 × Annexin. The apoptosis rate was detected by flow cytometry.

### Reverse transcription quantitative polymerase chain reaction (RT-qPCR)

Total RNA was extracted using TRIzol reagent (15,596,026, Invitrogen) and reverse transcribed into cDNA using Ncode ™ miRNA First-Strand cDNA Synthesis kit (Thermo Fisher Scientific Inc., Waltham, MA, USA). The synthesized cDNA was detected using Fast SYBR Green PCR kit (Applied Biosystems, Inc., Carlsbad, CA, USA). The primer sequences of miR-615 were synthesized by Sangon Biotech (Shanghai, China). Reverse primer was 5′-AGTTAAGAGTAGTGGGGAGATTAA-3′ and forward primer was 5′-AAATTTTTTTTCTTTATTTACCCC-3′.

### Methylation-specific PCR (MSP)

The methylation level of miR-615 promoter region in HCT-8 cells or tumor tissues was detected using DNA Methylation-Gold™ kit (D5005, Zymo Research, Irvine, CA, USA). The primer sequences of methylation reaction for MSP amplification were miR-615-MD (5′-GGGCGGAGGCGTTTTTTTC-3′) and miR-615-MR (5′-CGACCGAAAAAAAAAAAAACGAAAACCG-3′). The primer sequences of unmethylation reaction were miR-615-UD (5′-AAAGTTTTTTGTTTGGGTGGAGGTGTTTTTTTTG-3′) and miR-615-UR (5′-ACCCACAACCAAAAAAAAAAAAAACAAAAACCA-3′). The primer sequences of miR-615 were synthesized by Sangon Biotech. The purified DNA was added into CT transformation reagent for denaturation and bisulfate transformation. The reaction column was used for desulfurization and purification. The purified DNA was used for subsequent PCR reaction. PCR products were subjected to agarose gel electrophoresis. Image analysis was performed by gel electrophoresis imaging and analysis system (Thermo Fisher Scientific).

### Chromatin immunoprecipitation (ChIP) assay

HCT-8 cells were treated with 4% formaldehyde (Aladdin Biochemistry, Shanghai, China) (final formaldehyde concentration was 1%). The collected cells were broken by ultrasound and added with anti-Dnmt3b (ab2851, 1:50, Abcam Inc., Cambridge, MA, USA), anti-Dnmt1 (ab13537, 1:50, Abcam) and anti-Dnmt3a (ab2850, 1:50, Abcam) to bind the miR-615 gene promoter. Then, the cells were added with Protein A Agarose/Salmon Sperm DNA (Merck Millipore, Billerica, MA, USA) to bind to the promoter complex and precipitate the complex. The precipitated complex was cleaned to remove some nonspecific binding. After elution, the enriched miR-615 promoter complex was obtained and then crosslinked. The promoter fragment of enriched miR-615 was purified for qPCR.

### Methylated DNA immunoprecipitation (meDIP)

meDIP was performed using MeDIP kit (MSK Biotechnology Co., Ltd., Wuhan, Hubei, China). Briefly, genomic DNA was extracted from HCT-8 cells and purified using standard procedures. Genomic DNA was cut by ultrasound to produce 200–1000 BP random fragments. The DNA fragment was denatured at 95 °C to obtain single stranded DNA fragment, which was then incubated with 5-mC antibody (ab214727, Abcam) to precipitate the DNA containing 5-mC. The 5-mC was captured by magnetic beads. The 5-mC antibody pull-down DNA was extracted and purified by phenol/chloroform for real-time fluorescent quantitative PCR.

### Subcutaneous xenograft tumor in nude mice

Eighteen specific pathogen-free male BALB/c nude mice (5 weeks, 15–18 g) purchased from SLAC Laboratory Animal Co., Ltd. (Shanghai, China) were randomly assigned into control group, ^125^I group, ^125^I + miR-615 antagomir group, with 6 mice in each group. HCT-8 cells transfected with miR-615 antagomir were prepared into cell suspension (1 × 10^7^ cells/mL). The prepared cell suspension was injected into the left axillary skin of nude mice with a 1 mL syringe to establish the subcutaneous xenograft tumor model. When the tumor diameter of nude mice was about 1 cm, the ^125^I seeds were implanted into the tumor center. The mice in the ^125^I group were implanted with a ^125^I seed [0.8 mCi (29.97 MBq)] into the center of the tumor with a No. 18 implant needle. The mice in the ^125^I + miR-615 antagomir group were injected with miR-615 antagomir and implanted with 0.8 mCi ^125^I at a dose of 10 nmol/mouse. The mice were killed by cervical dislocation after 28 days of observation. The tumor tissues were dissected, photographed, weighed and measured. Some of the tumor tissues were used for DNA extraction and MSP.

### Immunohistochemistry

The tissues were fixed with 10% formaldehyde (Aladdin biochemistry), embedded in paraffin and sliced (4 μm). The tissue sections were dried in an oven at 60 °C for 1 h, dewaxed with conventional xylene (Aladdin biochemistry), then dehydrated with gradient alcohol, incubated in 3% H_2_O_2_ at 37 °C (Sigma-Aldrich) for 30 min, washed with PBS, boiled in 0.01 M citric acid buffer at 95 °C for 20 min, cooled to room temperature, and washed with PBS. The sections were blocked with normal goat serum working solution (Biolab Technology Co., Ltd., Beijing, China) at 37 °C for 10 min, and added with rabbit anti-Ki67 (1:500, ab15580, Abcam) at 4 °C for 12 h. After PBS washing, the sections were cultured with biotin-labeled goat anti-rabbit secondary antibody for 10 min. After washing, the sections were cultured with horseradish peroxidase-labeled streptomyces ovalbumin working solution (S-A/HRP) for 10 min. The sections were developed with 2,4-diaminobutyric acid (DAB) and stored in the dark for 8 min. Afterwards, the sections were washed with tap water, stained with hematoxylin violet, dehydrated, cleared, sealed, and observed under the light microscope. The positive cells were counted by Nikon image analysis software (Tokyo, Japan). Three visual fields (×200) were selected from each section to calculate the number of positive cells.

### TUNEL staining

The sections were dewaxed and treated with 50 μL 1% protease K (ST535, Beyotime Biotechnology Co., Ltd., Shanghai, China) diluent at 37 °C for 30 min. The sections were incubated with 0.3% H_2_O_2_ methanol solution at 37 °C for 30 min to eliminate endogenous peroxidase (POD) activity. Then the sections were incubated with TUNEL reaction solution (C1098, Beyotime) at 37 °C in the dark for 1 h and treated with 50 μL Converter-POD (C1098, Beyotime) at 37 °C for 30 min. Thereafter, the sections were developed with 2% DAB solution at room temperature for 15 min, followed by observation under the microscope. The reaction was terminated by adding distilled water after the brownish yellow nucleus appeared. The sections were counterstained with hematoxylin and the reaction was terminated by distilled water. The sections were dehydrated with 50, 70, 90 and 100% ethanol, cleared with xylene, sealed with neutral gum and observed under the microscope. Ten visual fields were randomly selected from each section. The cells with brown nuclei were apoptotic positive cells, and the blue nuclei were normal cells.

### Bioinformatics analysis

The enrichment of the KEGG pathway was analyzed using the KOBAS3.0 database (http://kobas.cbi.pku.edu.cn/kobas3/help/). The differential expressions of candidate target genes in colon and rectal cancer included in TCGA and GTEx were searched. The target genes of miR-615 were predicted using the TargetScan database (http://www.targetscan.org/vert_71/) and StarBase database.

### Statistical analysis

Data analysis was analyzed and introduced using SPSS 21.0 (IBM Corp., Armonk, NY, USA). Data are expressed as mean ± standard deviation. The *t* test was adopted for comparison between two groups. One-way analysis of variance (ANOVA) was employed for the comparisons among multiple groups, followed by Tukey’s multiple comparisons test. The *p* value was obtained from a two-tailed test, and the *p* < 0.05 meant a statistically significance.

## Results

### ^125^I seed irradiation inhibited growth and induced apoptosis of CRC cells *in vitro*

**Fig. 1:**
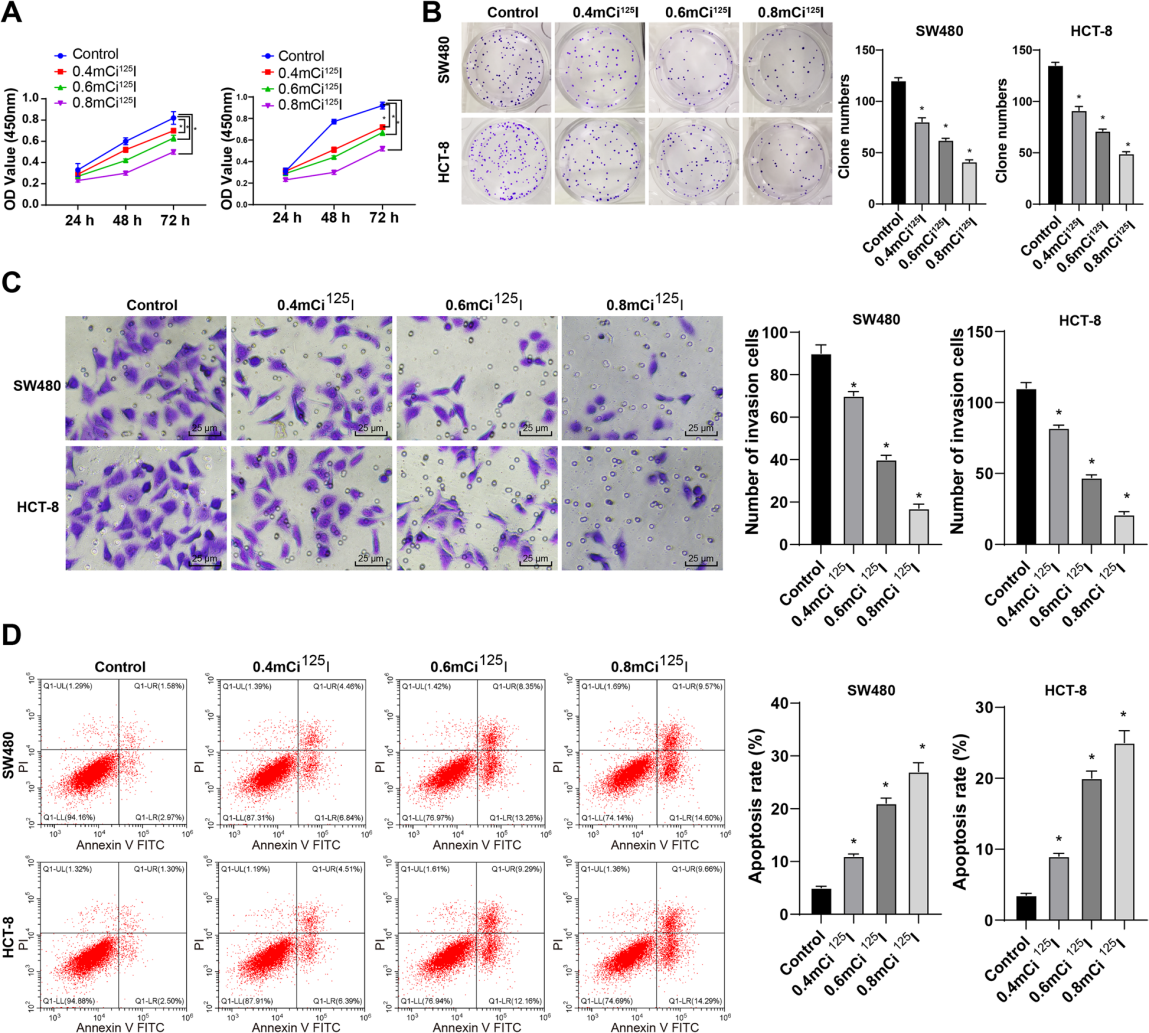
^125^I seed irradiation inhibited growth and induced apoptosis of CRC cells *in vitro*. **A**,**B** The cell growth in each group was measured using MTT assay and colony formation assay. **C** The cell invasion was measured using Transwell assay. **D** The cell apoptosis was detected using flow cytometry. The cell experiments were repeated 3 times independently. Data are presented as mean ± standard deviation and analyzed using one-way ANOVA, followed by Tukey’s multiple comparisons test. *p < 0.05

To explore the effect of ^125^I seed on CRC cells, we treated SW480 and HTC-8 cells with ^125^I seeds at the doses of 0.4 mCi (14.53 MBq), 0.6 mCi (22.79 MBq) and 0.8 mCi (29.97 MBq). MTT and colony formation assays showed that compared with that of the control cells, the growth rate of cells in the 0.4 mCi ^125^I, 0.6 mCi ^125^I and 0.8 mCi ^125^I groups was reduced with the increase of ^125^I irradiation dose (Fig. 1A-B). Transwell assay revealed that the invasion ability of cells in the 0.4 mCi ^125^I, 0.6 mCi ^125^I and 0.8 mCi ^125^I groups was decreased compared with that of the control cells (Fig. 1C). Flow cytometry showed that the cell apoptosis was notably increased in the 0.4 mCi ^125^I, 0.6 mCi ^125^I and 0.8 mCi ^125^I groups (Fig. 1D). Taken together, ^125^I seed irradiation inhibited the growth and promoted apoptosis of CRC cells *in vitro*, and the inhibitory effect was enhanced with the increase of radiation dose.

### miR-615 was poorly expressed in CRC, and overexpression of miR-615 inhibited the growth of CRC cells

**Fig. 2:**
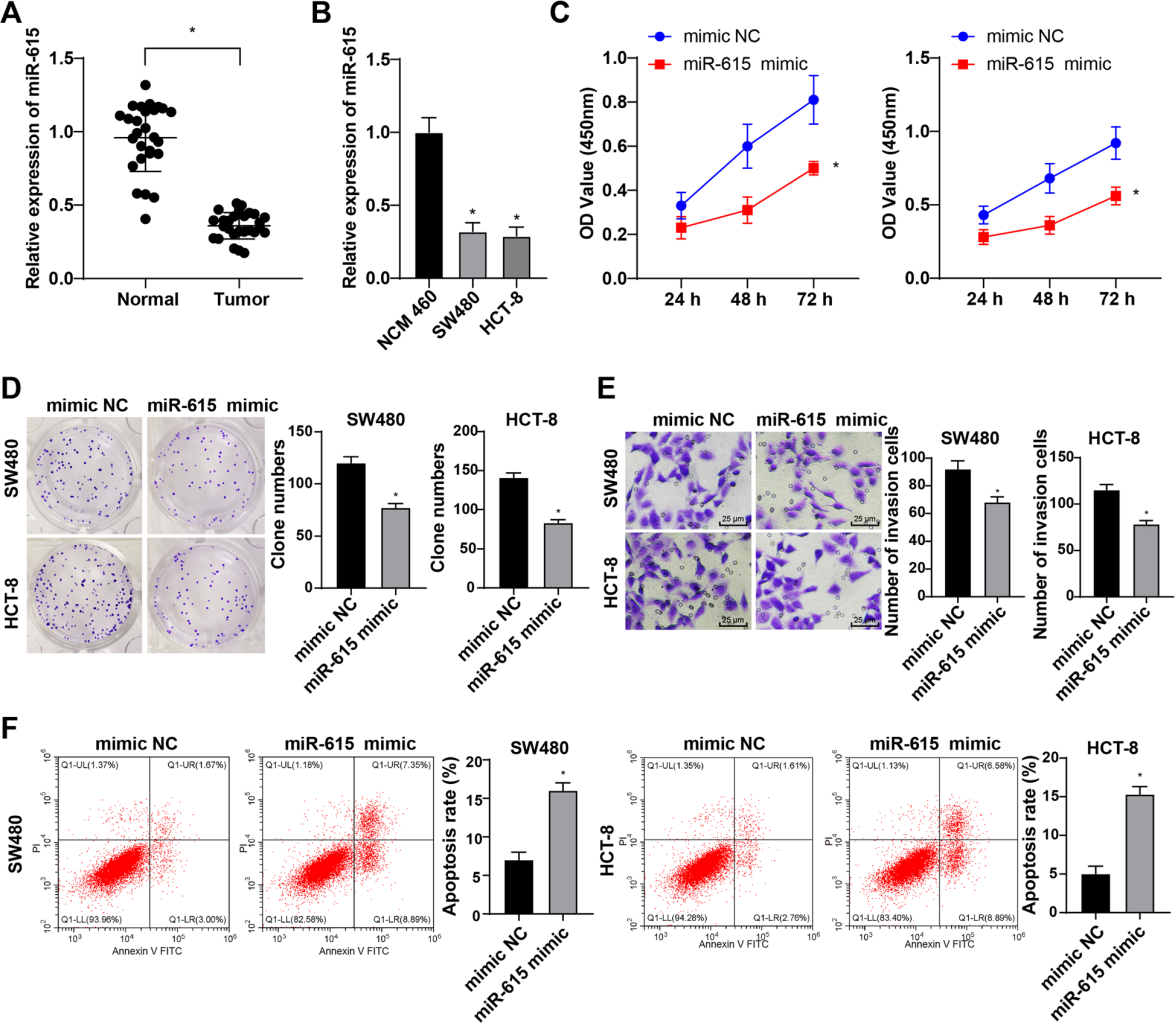
miR-615 was poorly expressed in CRC, and overexpression of miR-615 inhibited the growth of CRC cells. **A** miR-615 expression of normal colorectal tissues and CRC tissues was detected using RT-qPCR, N = 27. **B** miR-615 expression of normal colon epithelial cells and CRC cells was detected using RT-qPCR. **C**,**D** The cell growth in each group was measured using MTT assay and colony formation assay. **E** The cell invasion was measured using Transwell assay. **F** The cell apoptosis was detected using flow cytometry. The cell experiments were repeated 3 times independently. Data are presented as mean ± standard deviation. Data in panels A/D/E/F/G were analyzed using *t* test, and data in panel B and C were analyzed using one-way ANOVA, followed by Tukey’s multiple comparisons test. *p < 0.05 *vs.* the normal, NCM460 or mimic NC groups

miR-615 expression is notably downregulated in osteosarcoma and non-small cell lung cancer [19, 24]. To determine miR-615 expression in CRC, we detected the miR-615 expression of normal colorectal tissues and CRC tissues. The tumor tissues showed significantly reduced miR-615 expression compared with the normal tissues (Fig. 2A). Moreover, miR-615 expression of SW480 and HCT-8 cells was notably decreased compared with that of the NCM460 cells (Fig. 2B). The growth rate of miR-615 mimic-transfected cells was notably reduced compared with that of the mimic NC-transfected cells (Fig. 2C-D). The invasion ability of miR-615 mimic-transfected cells was decreased compared with that of the mimic NC-transfected cells (Fig. 2E). The cell apoptosis was notably increased in the 0.4 mCi miR-615 mimic group (Fig. 2F). Briefly, miR-615 was poorly expressed in CRC and overexpression of miR-615 inhibited growth and induced apoptosis of CRC cells.

### ^125^I seed inhibited the growth and induced apoptosis of CRC cells by activating miR-615 expression

**Fig. 3:**
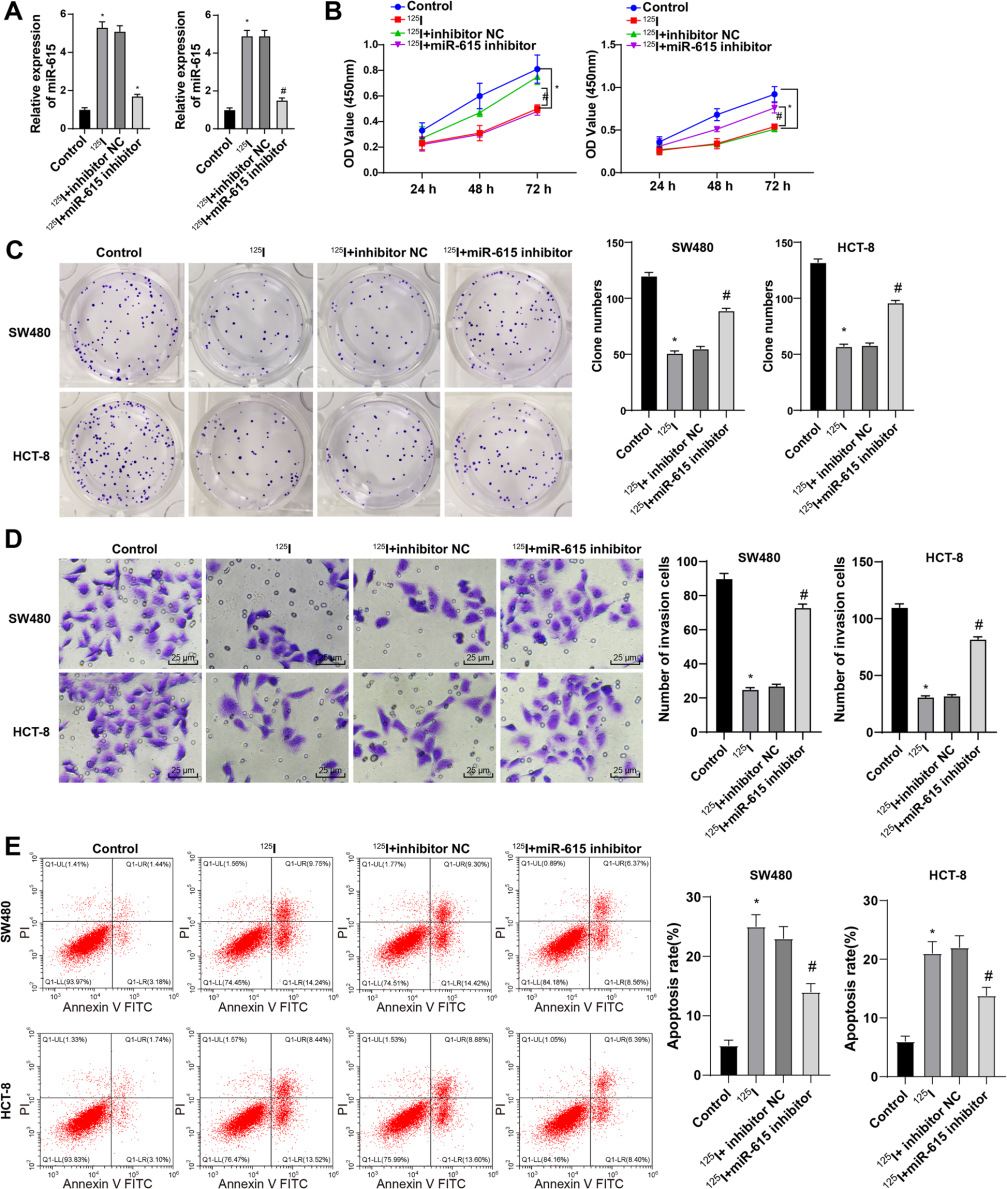
^125^I seed inhibited the growth and induced apoptosis of CRC cells by activating miR-615 expression. **A** miR-615 expression of cells in each group was detected using RT-qPCR. **B**,**C** The cell growth in each group was measured using MTT assay and colony formation assay. **D** The cell invasion was measured using Transwell assay. **E** The cell apoptosis was detected using flow cytometry. The cell experiments were repeated 3 times independently. Data are presented as mean ± standard deviation. Data were analyzed using one-way ANOVA, followed by Tukey’s multiple comparisons test. *p < 0.05 *vs.* the control group; #*p* < 0.05 *vs.* the ^125^I + inhibitor NC group

CRC cells were treated with ^125^I seed at a dose of 0.8 mCi (29.97 MBq) in the following experiments, named ^125^I group. The 0.8 mCi ^125^I-treated cells were transfected with miR-615 inhibitor. Compared with the control group, the ^125^I group showed increased miR-615 expression; compared with the ^125^I + inhibitor NC group, the ^125^I + miR-615 inhibitor group showed notably reduced miR-615 expression (Fig. 3A). Compared with the control group, the ^125^I group had decreased cell growth rate; compared with the ^125^I + inhibitor NC group, the ^125^I + miR-615 inhibitor group showed notably enhanced cell growth rate (Fig. 3B-C). Compared with the control group, the ^125^I group had decreased invasion ability; compared with the ^125^I + inhibitor NC group, the ^125^I + miR-615 inhibitor group showed notably enhanced invasion ability (Fig. 3D). Compared with the control group, the ^125^I group had increased cell apoptosis; compared with the ^125^I + inhibitor NC group, the ^125^I + miR-615 inhibitor group showed notably decreased cell apoptosis (Fig. 3E). Briefly, ^125^I seed inhibited the growth and induced apoptosis of CRC cells by activating miR-615 expression.

### ^125^I seed activated miR-615 expression by inducing demethylation of miR-615 promoter

**Fig. 4:**
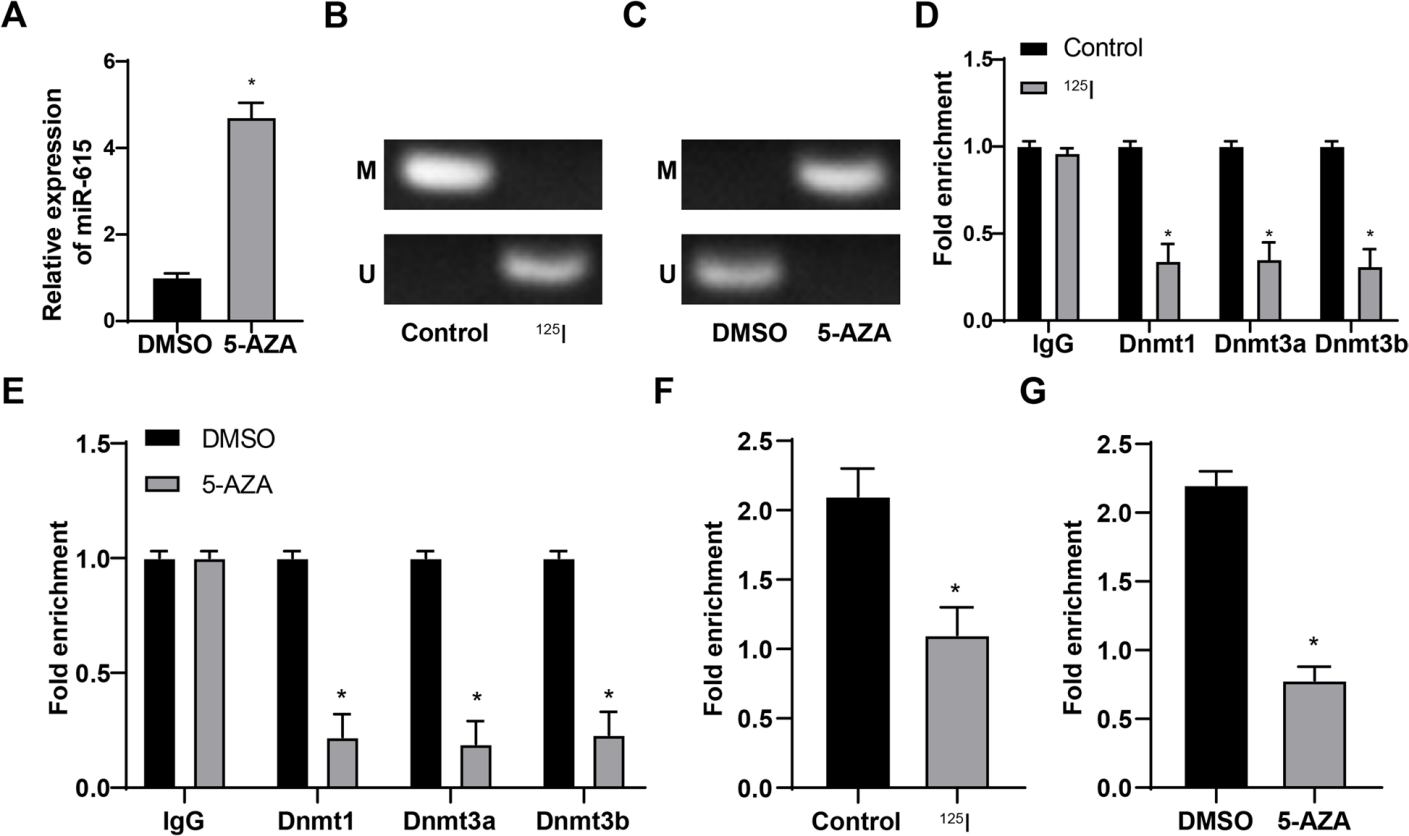
^125^I seed activated miR-615 expression by inducing demethylation of miR-615 promoter. **A** miR-615 expression of cells in each group was detected using RT-qPCR. **B**,**C** The methylation level of miR-615 promoter was detected using MSP. **D**,**E** The enrichment of DNMT1, DNMT3a and DNMT3b in miR-615 promoter region was measured using ChIP, and IgG acted as the negative control. **F**,**G** The methylation level of miR-615 promoter was detected using meDIP. The cell experiments were repeated 3 times independently. Data are presented as mean ± standard deviation. Data in panels A/F/G were analyzed using *t* test, and data in panels D/E were analyzed using one-way ANOVA, followed by Tukey’s multiple comparisons test. *p < 0.05 *vs.* the control or DMSO groups

^125^I seed can modulate miR expression by affecting its methylation [25]. High methylation level of miR-615 inhibits its expression and promotes tumor growth and metastasis [20]. To explore whether ^125^I seed regulated the methylation of miR-615 in CRC, we detected miR-615 expression in HCT-8 cells treated with ^125^I or 5-AZA. Compared with the control group, the ^125^I group showed increased miR-615 expression (Fig. 4A); compared with the DMSO group, the 5-AZA group showed notably increased miR-615 expression (Fig. 4A). The methylation of miR-615 promoter was detected using MSP. Compared with the control group, the ^125^I group showed decreased methylation at specific CpG site; compared with the DMSO group, the 5-AZA group showed increased methylation at specific CpG site (Fig. 4B-C). ChIP was used to measure the enrichment of DNMT1, DNMT3a and DNMT3b in miR-615 promoter region. Compared with the control group, the ^125^I group showed decreased enrichment of DNMT1, DNMT3a and DNMT3b in miR-615 promoter region; compared with the DMSO group, the 5-AZA group showed reduced enrichment of DNMT1, DNMT3a and DNMT3b in miR-615 promoter region (Fig. 4D-E). The methylation level of miR-615 promoter was detected using meDIP assay. Compared with the control group, the ^125^I group showed decreased methylation level of miR-615 promoter region; compared with the DMSO group, the 5-AZA group showed reduced methylation level of miR-615 promoter region (Fig. 4F-G). Taken together, ^125^I seed activated miR-615 expression by inducing demethylation of miR-615 promoter.

### ^125^I seed inhibited the growth of CRC *in vivo* by activating miR-615 expression

**Fig. 5:**
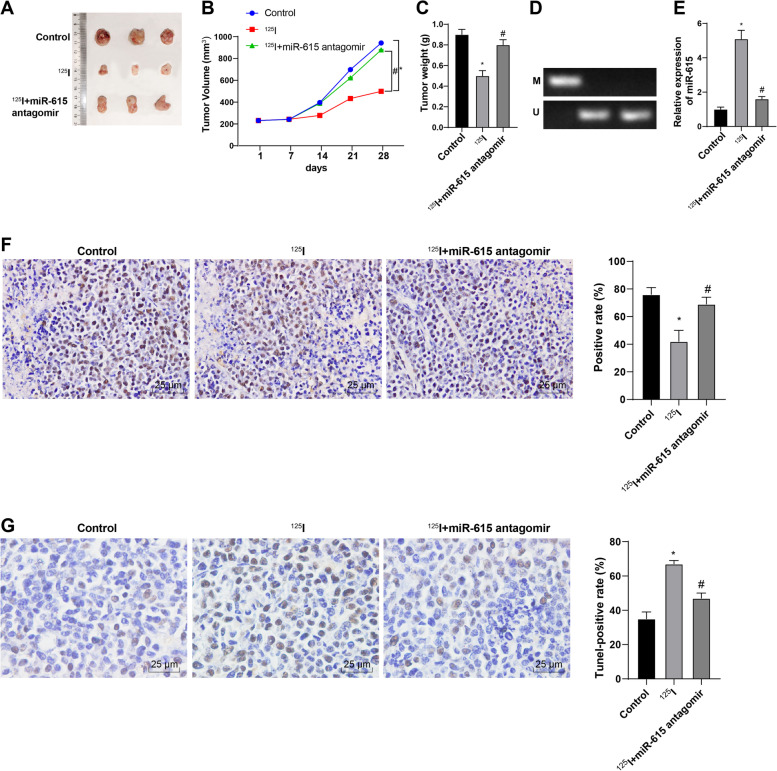
^125^I seed inhibited the growth of CRC cells *in vivo* by activating miR-615 expression. **A** Images of tumors in each group. **B** Tumor growth curve. **C** Tumor weight. **D** The methylation level of miR-615 in tumors was detected using MSP. **E** miR-615 expression of cells in each group was detected using RT-qPCR. **F** The expression of Ki67 was detected using immunohistochemistry. **G** Apoptosis of tissues in each group was detected using TUNEL. N = 3. The cell experiments were repeated 3 times independently. Data are presented as mean ± standard deviation. Data were analyzed using one-way ANOVA, followed by Tukey’s multiple comparisons test. *p < 0.05 *vs.* the control group; #*p* < 0.05 *vs.* the ^125^I group

To investigate the effects of ^125^I and miR-615 on CRC *in vivo*, we treated mice with miR-615 antagomir and 0.8 mCi ^125^I. Compared with that of the control mice, the tumor growth of ^125^I-treated mice was notably decreased; compared with that of the ^125^I-treated mice, the tumor growth of ^125^I + miR-615 antagomir-treated mice was increased (Fig. 5A-C). Compared with the control group, the ^125^I group showed decreased methylation of miR-615 and increased miR-615 expression; compared with the ^125^I group, the ^125^I + miR-615 antagomir group showed no significant difference in methylation of miR-615 but notably reduced miR-615 expression (Fig. 5D,E). Compared with the control group, the ^125^I group showed decreased Ki67 expression; compared with the ^125^I group, the ^125^I + miR-615 antagomir group showed increased Ki675 expression (Fig. 5F). Compared with the control group, the ^125^I group showed enhanced apoptosis; compared with the ^125^I group, the ^125^I + miR-615 antagomir group had reduced apoptosis (Fig. 5G). Briefly, ^125^I seed inhibited the growth and induced apoptosis of CRC *in vivo* by inhibiting miR-615 promoter methylation.

### Prediction of miR-615-5p target gene and bioinformatics analysis of related pathways

**Fig. 6:**
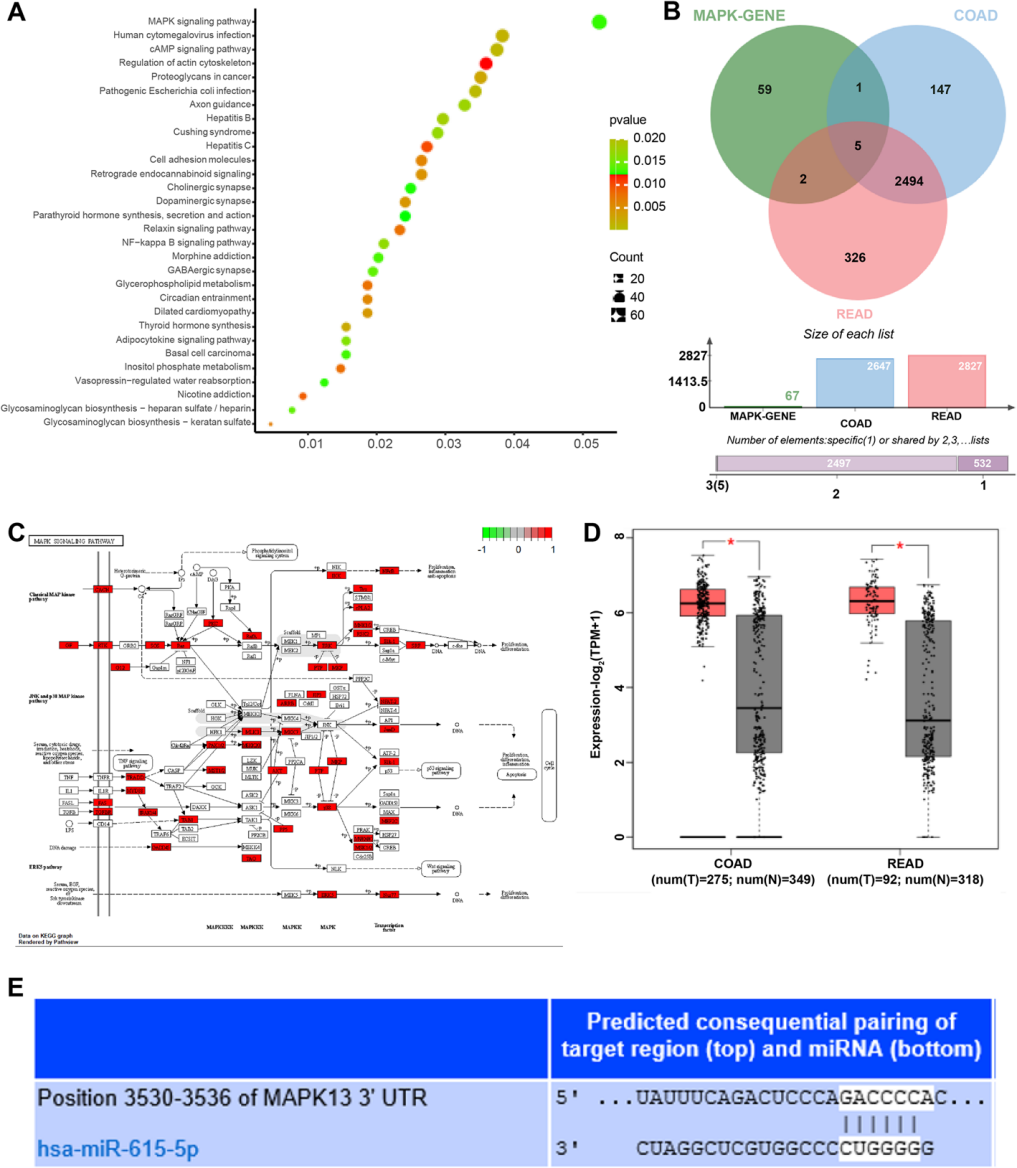
Bioinformatics predicted that miR-615-5p regulated MAPK13 expression and affected the MAPK pathway, thus affecting the progression of CRC. **A** The enrichment of miR-615-5p target genes was analyzed; **B**-**C** The intersection of the candidate target genes in the MAPK pathway and the overexpressed genes in colon and rectal cancer in TCGA and GTEx was taken; **D** MAPK13 expression in CRC was analyzed using the TCGA database; **E** miRbase predicted the binding sites between miR-615-5p and MAPK13

The enrichment analysis of miR-615-5p target gene elicited that the target genes were mainly enriched in the MAPK pathway (Fig. 6A). The intersection of the candidate target genes in the MAPK pathway and the overexpressed genes in colon and rectal cancer in TCGA and GTEx was taken, and 5 candidate genes EFNA2, RPS6KA2, MAPK13, EFNA3 and FGFR3 were identified. Among these, MAPK (p38) was at the core of the MAPK pathway (Fig. 6B-C, Supplementary Table 2, 3). MAPK13 was significantly overexpressed in colon and rectal cancer (Fig. 6D). Meanwhile, miRBase predicted that there were binding sites between miR-615-5p and MAPK13 (Fig. 6E). Therefore, we proposed the future research direction that miR-615-5p targeted MAPK13 and affected the MAPK pathway, thus affecting the progression of CRC.

## Discussion

CRC mostly develops from benign polyps to distant metastasis, and consequently, early diagnosis and intervention are essential for prolonging the long-term survival [26]. ^125^I seed implantation has emerged as a safe treatment modality for human malignancies, including CRC [6, 10]. This study demonstrated that ^125^I seed implantation exerted therapeutic effects on CRC by modulating miR-615 gene methylation.

Accumulating evidence has identified that ^125^I seed implantation can serve as a feasible and effective salvage or palliative treatment modality for patients suffering from recurrent or metastatic CRC [10, 27, 28]. However, the specific mechanism and biological effects of ^125^I seed implantation on CRC cells were still largely undetermined. To explore the effect of ^125^I seed on CRC, we treated CRC cells with ^125^I seeds of 0.4 mCi, 0.6 mCi and 0.8 mCi. Then, the growth and apoptosis of the ^125^I seed-treated CRC cells were measured. The destruction of cell cycle checkpoint control and the impaired balance between proliferation and apoptosis are vital attributes resulting in tumor progression [27]. In this study, ^125^I seed irradiation inhibited the growth and promoted apoptosis of CRC cells *in vitro*, and the inhibitory effect was enhanced with the increase of radiation dose. Consistently, Ma et al. have revealed that ^125^I triggers CRC cell apoptosis by increasing p53 and decreasing vascular endothelial growth factor [29].

Emerging evidence has unveiled that ^125^I seed represses the tumor progression and metastasis by modulating miR expression [25, 30]. miRNAs are is deeply implicated in tumor initiation and progression, either functioning as a tumor promoter or a tumor suppressor [31]. miR-615 is identified to serve as a tumor suppressor in renal cell carcinoma and osteosarcoma [19, 32]. Still, the exact role of miR-615 in CRC remained controversial. This study demonstrated that miR-615 was notably downregulated in CRC tissues and cells. Then the CRC cells were transfected with miR-615 mimic, and the results revealed that overexpression of miR-615 inhibited growth and induced apoptosis of CRC cells. Wu et al. have demonstrated that overexpression of hsa_circRNA_002144 facilitates CRC progression via sponging miR-615-5p [33], implying the role of miR-615 as a CRC intervention target. Moreover, the ^125^I seed-treated CRC cells were transfected with miR-615 inhibitor. After ^125^I + miR-615 inhibitor treatment, the growth rate of CRC cells was increased and the apoptosis was reduced notably. Taken together, ^125^I seed inhibited the growth and induced apoptosis of CRC cells by activating miR-615 expression.

Aberrant DNA methylation is critically implicated in the deregulation of miRs in cancers leading to the tumor initiation and progression [12, 13]. The alteration of DNA methylation pattern plays a crucial role in tumor suppression induced by low-energy ^125^I irradiation [34]. miR-615-5p is hypermethylated in pancreatic ductal adenocarcinoma cells, thus resulting in tumor growth and invasion [20]. It was reasonable to assume that miR-615 was reactivated in CRC cells by ^125^I irradiation-induced demethylation, thus exerting its anticancer effect. DNA methylation can affect CpG island, which leads to transcriptional silence by affecting transcription factor binding and chromatin structure changes [35]. The DNA methyltransferases (DNMT1, DNMT3a and DNMT3b) are major functional enzymes in mammalian cells to establish and maintain DNA methylation pattern [12]. Consecutive low-energy ^125^I irradiation can notably suppress the levels of DNA methyltransferases in cancer cells [12]. In the current study, the ^125^I seed or 5-AZA-treated CRC cells showed decreased methylation at specific CpG site and reduced enrichment of DNMT1, DNMT3a and DNMT3b in the miR-615 promoter. These results confirmed that miR-615 expression in CRC cells was enhanced and the miR-615 promoter methylation was reduced after ^125^I seed or 5-AZA treatment. In brief, ^125^I seed activated miR-615 expression by inducing demethylation of miR-615 promoter. *In vivo* experiments revealed that the ^125^I + miR-615 antagomir-treated mice had decreased miR-615 expression, enhanced tumor growth and Ki67 expression, and reduced apoptosis of CRC cells. It was verified that ^125^I seed inhibited the growth and induced apoptosis of CRC cells *in vivo* by inhibiting miR-615 promoter methylation. Furthermore, bioinformatics analysis elicited that the miR-615-5p target genes were mainly enriched in the MAPK pathway. We took the intersection of the candidate target genes in the MAPK pathway and the overexpressed genes in colon and rectal cancer in TCGA and GTEx and obtained MAPK13, which was at the core in the MAPK pathway and clearly elevated in CRC. The binding sites between miR-615-5p and MAPK13 were predicted. Consistently, MAPK13 plays a pro-oncogenic role in colitis-associated CRC [36]. In conclusion, miR-615-5p targeted MAPK13 and affected the MAPK pathway, thus affecting the progression of CRC.

To sum up, ^125^I seed repressed the growth and facilitated apoptosis of CRC cells by suppressing the methylation of miR-615 promoter and activating miR-615 expression. Collectively, ^125^I irradiation-induced CRC cell apoptosis and DNA demethylation might be two pivotal mechanisms in the therapeutic effect of ^125^I seed implantation. However, our current study showed that ^125^I particles activated the expression of miR-615 by inhibiting the methylation of miR-615 promoter, thus inhibiting the growth of CRC cells *in vivo* and *in vitro*, and promoting their apoptosis. Due to the impact of epidemic situation, experimental funds, experimental conditions and other reasons, the downstream target genes of miR-615 have not been explored at present. We are very interested in this downstream direction and make it the goal of our future exploration. If the experimental conditions permit in the future, we will carry out relevant research. In addition, this study preliminarily identified the expression of miR-615 in CRC, and its specific effects and downstream mechanism remained further exploration. In  future research, we shall investigate the complete mechanism of miR-615 in the effect of ^125^I seed implantation on CRC.

## Supplementary Information

**Additional file 1: Supplementary Table 1** Clinical data of patients.

**Additional file 2..**

**Additional file 3..**

**Additional file 4..**

## Data Availability

All the data generated or analyzed during this study are included in this published article.
