# Supplementary material for: Iodine-125 seed represses the growth and facilitates the apoptosis of colorectal cancer cells by suppressing the methylation of miR-615 promoter
Source: BMC Cancer. 2022 Jan 8;22:49. doi: 10.1186/s12885-021-09141-4 (PMC8742920; doi:10.1186/s12885-021-09141-4)
Supplement: Supplementary file 1 — Additional file 1: Supplementary Table 1 Clinical data of patients. [file 12885_2021_9141_MOESM1_ESM.docx]

**Supplementary Table 1** Clinical data of patients

| Clinical features | Patient number N = 27 |
| --- | --- |
| Gender |  |
| Male | 14 |
| Female | 13 |
| Age |  |
| < 45 | 3 |
| 45-55 | 6 |
| 55-65 | 7 |
| > 65 | 11 |
| CRC type |  |
| Colon cancer | 17 |
| Rectal cancer | 10 |
| Stool routine occult blood index |  |
| Positive | 15 |
| Negative | 12 |
