# Supplementary figures and images for: Iodine-125 seed represses the growth and facilitates the apoptosis of colorectal cancer cells by suppressing the methylation of miR-615 promoter
Source: BMC Cancer. 2022 Jan 8;22:49. doi: 10.1186/s12885-021-09141-4 (PMC8742920; doi:10.1186/s12885-021-09141-4)

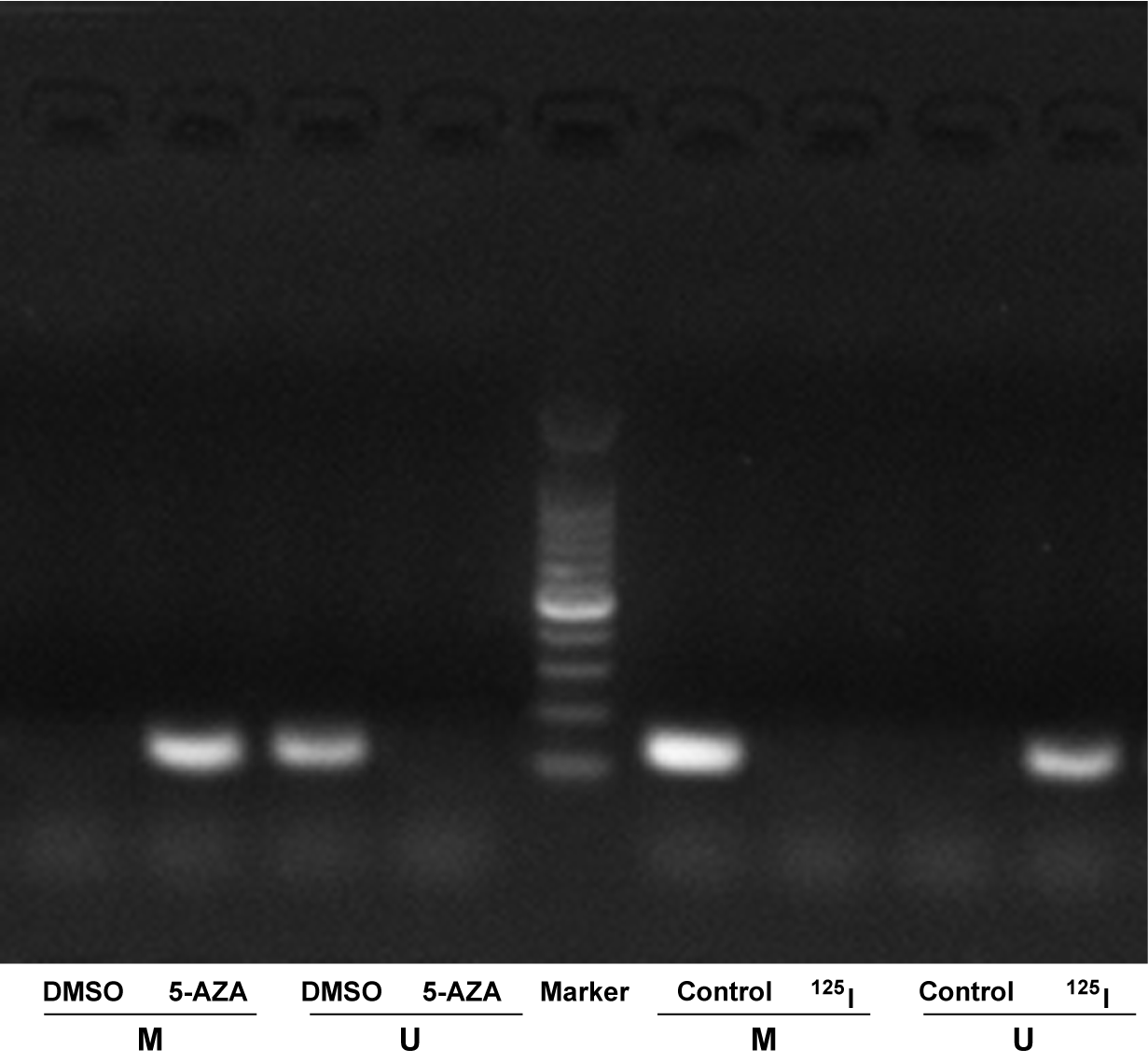

Supplement: Supplementary file 4 — Additional file 4.. [file 12885_2021_9141_MOESM4_ESM.zip › 4B & 4C.tiff]

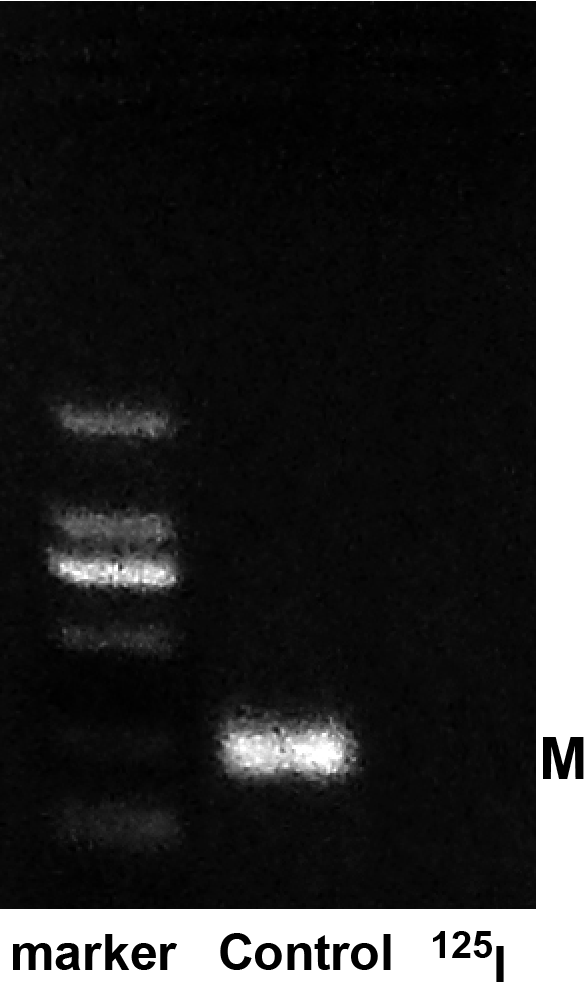

Supplement: Supplementary file 4 — Additional file 4.. [file 12885_2021_9141_MOESM4_ESM.zip › 4B M.tiff]

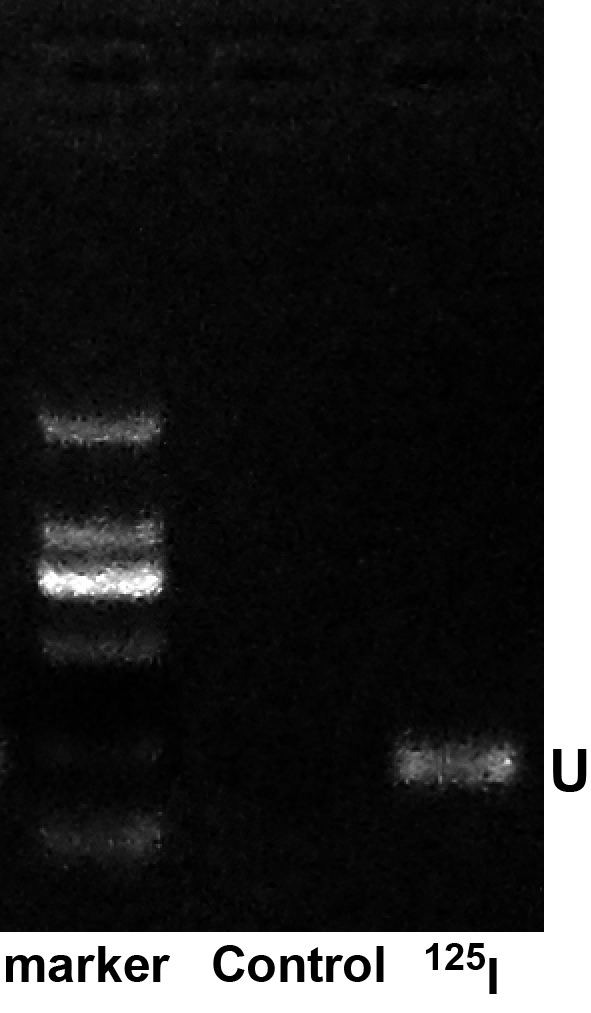

Supplement: Supplementary file 4 — Additional file 4.. [file 12885_2021_9141_MOESM4_ESM.zip › 4B U.tiff]

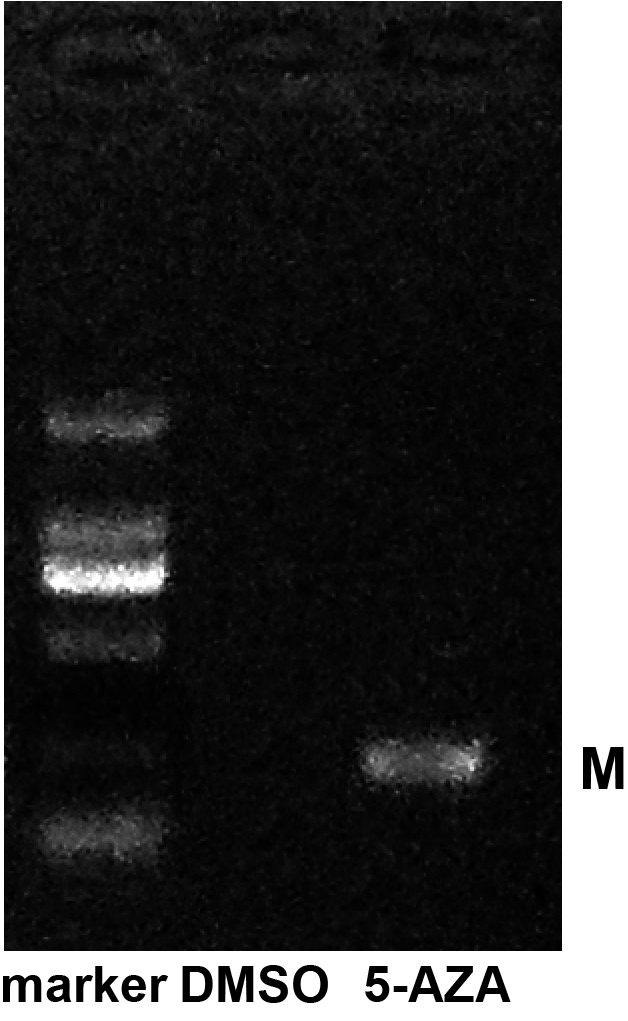

Supplement: Supplementary file 4 — Additional file 4.. [file 12885_2021_9141_MOESM4_ESM.zip › 4C M.tiff]

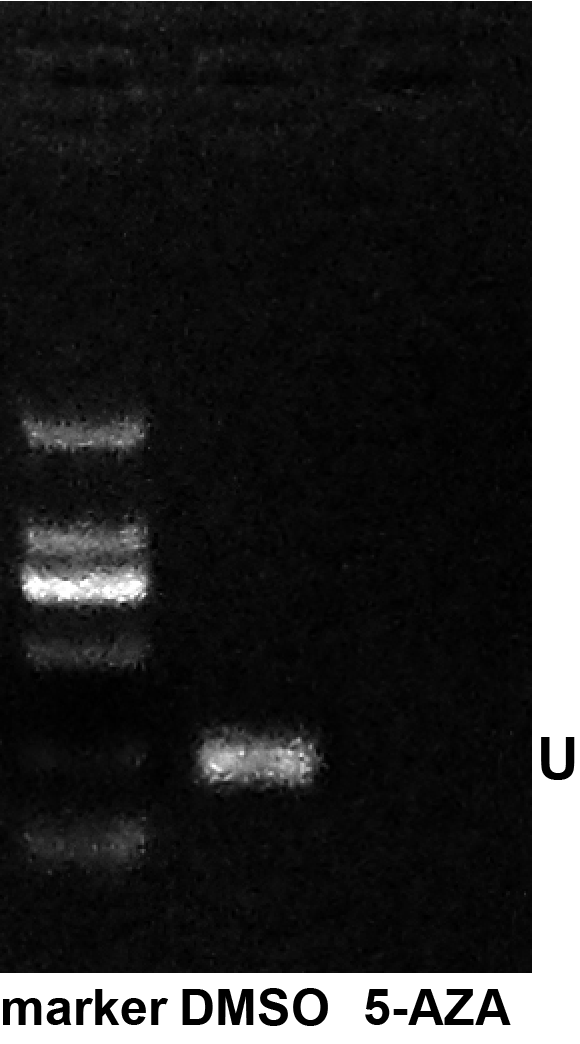

Supplement: Supplementary file 4 — Additional file 4.. [file 12885_2021_9141_MOESM4_ESM.zip › 4C U.tiff]

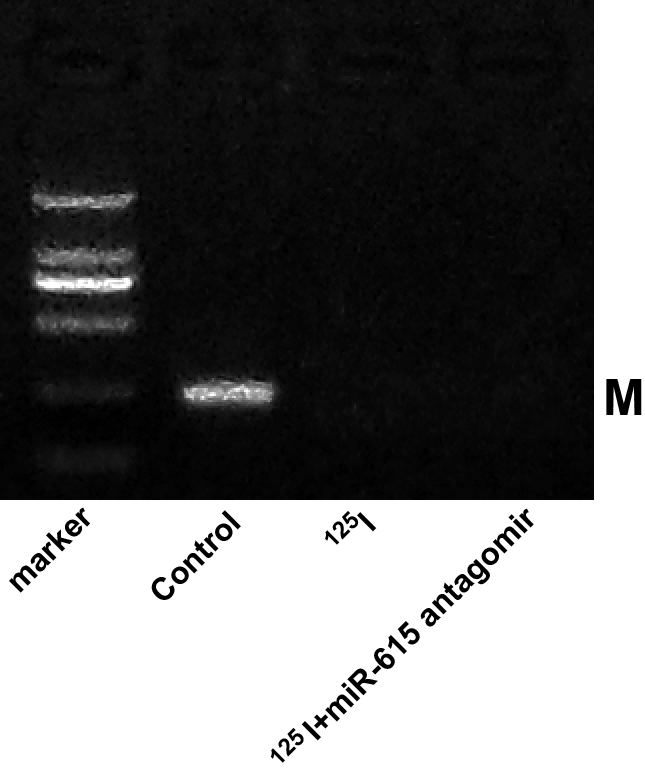

Supplement: Supplementary file 4 — Additional file 4.. [file 12885_2021_9141_MOESM4_ESM.zip › 5D M.tiff]

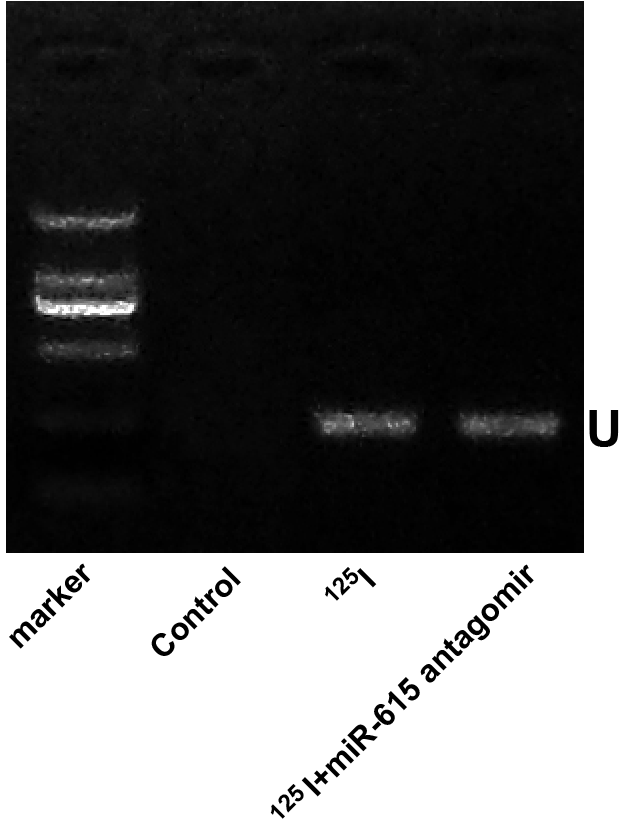

Supplement: Supplementary file 4 — Additional file 4.. [file 12885_2021_9141_MOESM4_ESM.zip › 5D U.tiff]

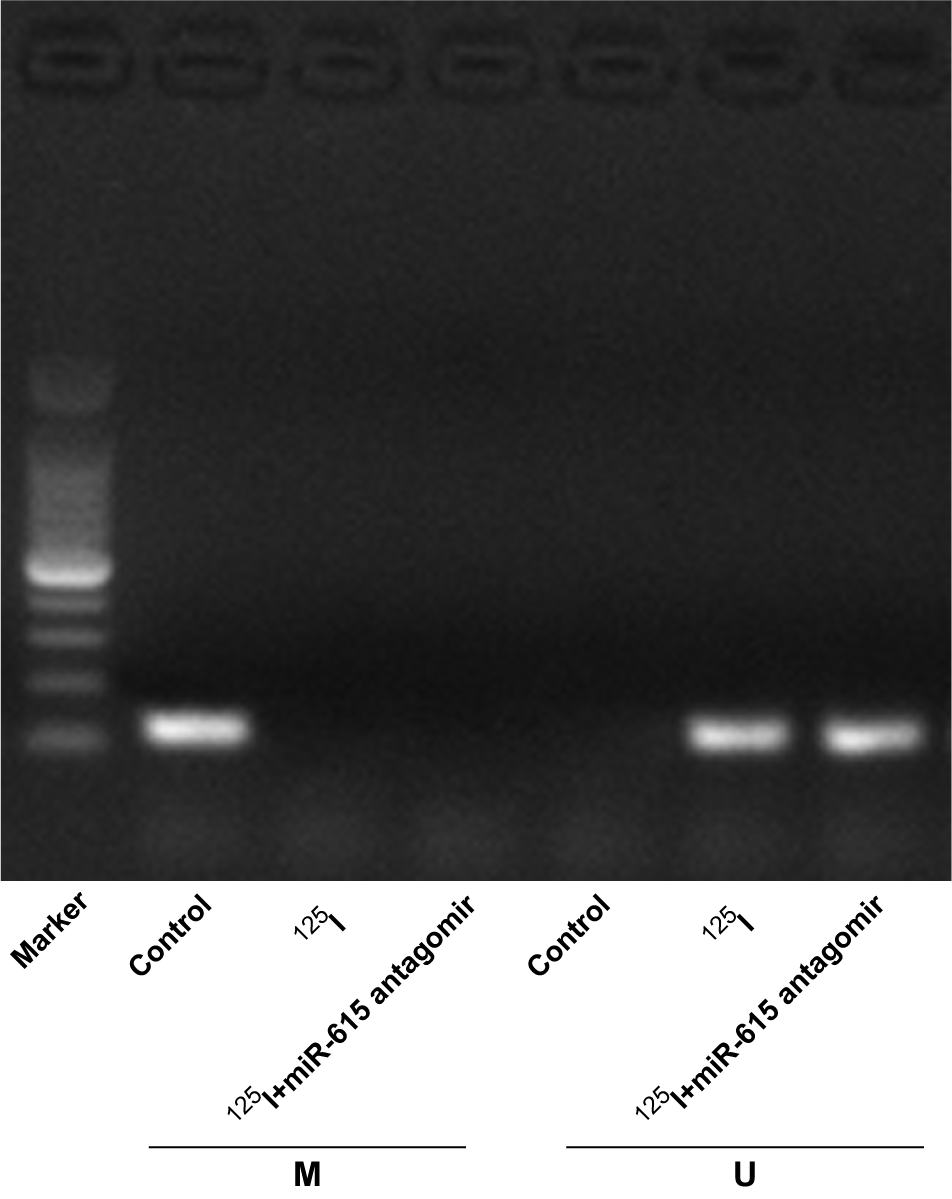

Supplement: Supplementary file 4 — Additional file 4.. [file 12885_2021_9141_MOESM4_ESM.zip › 5D.tiff]
